# Transverse Strips Instead of Wearable Laser Lights Alleviate the Sequence Effect Toward a Destination in Parkinson's Disease Patients With Freezing of Gait

**DOI:** 10.3389/fneur.2020.00838

**Published:** 2020-08-12

**Authors:** Shan-Shan Cao, Xiang-Zhen Yuan, Shu-Hong Wang, Reyisha Taximaimaiti, Xiao-Ping Wang

**Affiliations:** Department of Neurology, Tongren Hospital, Shanghai Jiao Tong University School of Medicine, Shanghai, China

**Keywords:** Parkinson's disease, freezing of gait, sequence effect, destination, visual cues, transverse strips, laser lights, rehabilitation

## Abstract

**Background:** The sequence effect (SE), referring to step-to-step reduction in amplitude, is considered to lead to freezing of gait (FOG) in Parkinson's disease (PD). Visual cues may alleviate SE and help reduce freezing episodes. FOG patients show significant SE prior to turning or toward a doorway, but the SE toward a destination has not been clearly studied.

**Objectives:** To examine the SE when approaching a destination in PD patients with FOG, and to further explore the effects of different types of visual cues on destination SE.

**Methods:** Thirty-five PD patients were divided into a freezing (PD+FOG, *n* = 15) group and a non-freezing (PD–FOG, *n* = 20) group. Walking trials were tested under three conditions, including without cues (no-cue condition), with wearable laser lights (laser condition), and with transverse strips placed on the floor (strip condition). Kinematic data was recorded by a portable Inertial Measurement Unit (IMU) system. The destination SE and some key gait parameters were evaluated.

**Results:** The PD+FOG group showed greater destination SE in the no-cue and laser conditions when compared to the PD–FOG group. There were no significant differences in the strip condition when comparing destination SE of the two groups. The destination SE was alleviated only by using the transverse strips on the floor. In contrast, transverse strips and wearable laser lights could increase the step length.

**Conclusions:** The significant destination SE may explain why FOG patients are prone to freezing when heading toward their destination. Visual cues using transverse strips on the floor may be a more effective strategy for FOG rehabilitation in PD patients.

## Introduction

Freezing of gait (FOG), defined as “a brief, episodic absence or a marked reduction of forward progression of the feet despite the intention to walk” (1), is a debilitating symptom in Parkinson's disease (PD). The incidence and severity of FOG increase as the disease progresses (2). FOG is dramatically influenced by environmental factors and tends to occur when turning, passing through a doorway or approaching a destination (3). In addition, medication state (off or on L-dopa condition), cognitive overload and negative emotions (anxiety or depression) can also precipitate FOG (4–7). Due to its paroxysmal and unpredictable features, FOG can easily cause falls and increase the risk of fractures, thus further causing worse prognosis and increasing the burden on families and society (8, 9).

However, the pathophysiology of FOG remains unclear (4). The progressively decreasing step length has been reported in steps prior to freezing (10). The phenomenon of gradual step to step reduction is termed sequence effect (SE), which may attribute to the inability of basal ganglia (BG) to provide timing cues and is believed to cause FOG in PD patients (11, 12). Based on the defects of BG function and gait-control system, the concept of dual requirement of background step length reduction (manifestation of gait hypokinesia) and presence of SE can explain most of the freezing phenomenon in PD (13). Chee et al. reported that FOG episodes were induced more frequently through voluntarily diminishing step length if a significant SE was co-existent in the PD patient (14). Particularly, motor blocks will not occur in the absence of SE during walking (13).

The severity of SE is influenced by environmental factors and varies between individuals, therefore the SE can be much greater under some circumstances (13). For example, it has been shown that prior to turning, the SE in PD patients with FOG was significantly greater than that in healthy people and PD patients without FOG, although all groups perform progressive step-to-step reduction (15). These could partly explain why turning induces freezing episodes in PD. Another study explored the gait changes of participants when they walked through a variable-width doorway. PD group had greater gait changes and their step length decreased significantly when approaching the narrow doorway (16). If the SE attended, it could result in a motor block. In fact, destination freezing is also one of common types of FOG in PD patients (3). Although reducing step length is an expectable reaction to the approaching destination, SE toward a destination has not been directly demonstrated in PD patients.

The treatment of FOG still poses a clinical challenge (17, 18). Therefore, alleviating the SE may provide a new therapeutic option for FOG in PD. Iansek et al. investigated the SE in FOG patients and found that the SE was eliminated by using visual cues, but it did not respond to L-dopa or attention strategies (11). In that study, they chose transverse white strips on the floor as visual cues. However, it remains unclear whether other types of visual cues (e.g., wearable laser lights) could alleviate SE in a similar way.

The purpose of this study is to compare the SE toward a destination between PD patients with and without FOG and evaluate the effects of two types of visual cues (transverse strips on the floor and wearable laser lights) on the destination SE and some key gait parameters.

## Materials and Methods

### Participants

A total of 35 participants with idiopathic PD were recruited from the Movement Disorders Clinic at Tongren Hospital, Shanghai Jiao Tong University School of Medicine, including 15 patients with FOG (the PD+FOG group) and 20 patients without FOG (the PD–FOG group). All participants were diagnosed in terms of the MDS Clinical Diagnostic Criteria for Parkinson's Disease. Participants were included if they could independently walk a 10-m distance for several times, with periodical rest. Exclusion criteria included any additional brain parenchyma injuries (e.g., stroke, hydrocephalus, brain tumors or traumatic brain injury), ophthalmic or orthopedic conditions that might affect gait, and cognitive deficits that cannot complete the experiment. PD patients were identified experiencing FOG, if they scored 1 “I have experienced such a feeling or episode over the past month” on Part I question of New Freezing of Gait Questionnaire (NFOG-Q) (19) or if they were detected freezing in the outpatient clinic.

This study was approved by the Medical Ethics Committee of TongRen Hospital, Shanghai Jiao Tong University School of Medicine. Written informed consent was obtained from all patients prior to testing.

### Clinical Assessments

In the dopaminergic “on” state, demographic data (e.g., age, gender, height and disease duration) of each subject was collected, and clinical assessments were evaluated. Motor performance was assessed with Part III of the Unified Parkinson's Disease Rating Scale (UPDRS-III). Cognitive and affective conditions were evaluated with Mini-Mental State Examination (MMSE), Montreal Cognitive Assessment-Basic (MoCA-B) Chinese Version and Geriatric Depression Scale (GDS). Subjective severity of FOG was assessed using the NFOG-Q. The other clinical variables included the Hoehn and Yahr (H&Y) scale for evaluating disease severity and 39-item Parkinson's Disease Questionnaire (PDQ-39) for assessing quality of life.

### Equipment and Gait Protocol

To measure spatiotemporal gait parameters, a portable Inertial Measurement Unit (IMU) system (GYENNO Science, Shenzhen, China) was applied, with 10 inertial sensors placed on each subject's lower back, chest, and bilateral feet, ankles, thighs and wrists by elastic belts. Each sensor collected spatiotemporal gait information in real time while the participants were walking, and transmitted the information to the host computer via a Bluetooth link for further processing and storage. IMU-based measurements can measure the fundamental gait parameters with sufficient accuracy in both healthy subjects and PD patients (20). The gait assessments were conducted in a hall with enough space to avoid environmental factors that might contribute to FOG.

**Figure 1:**
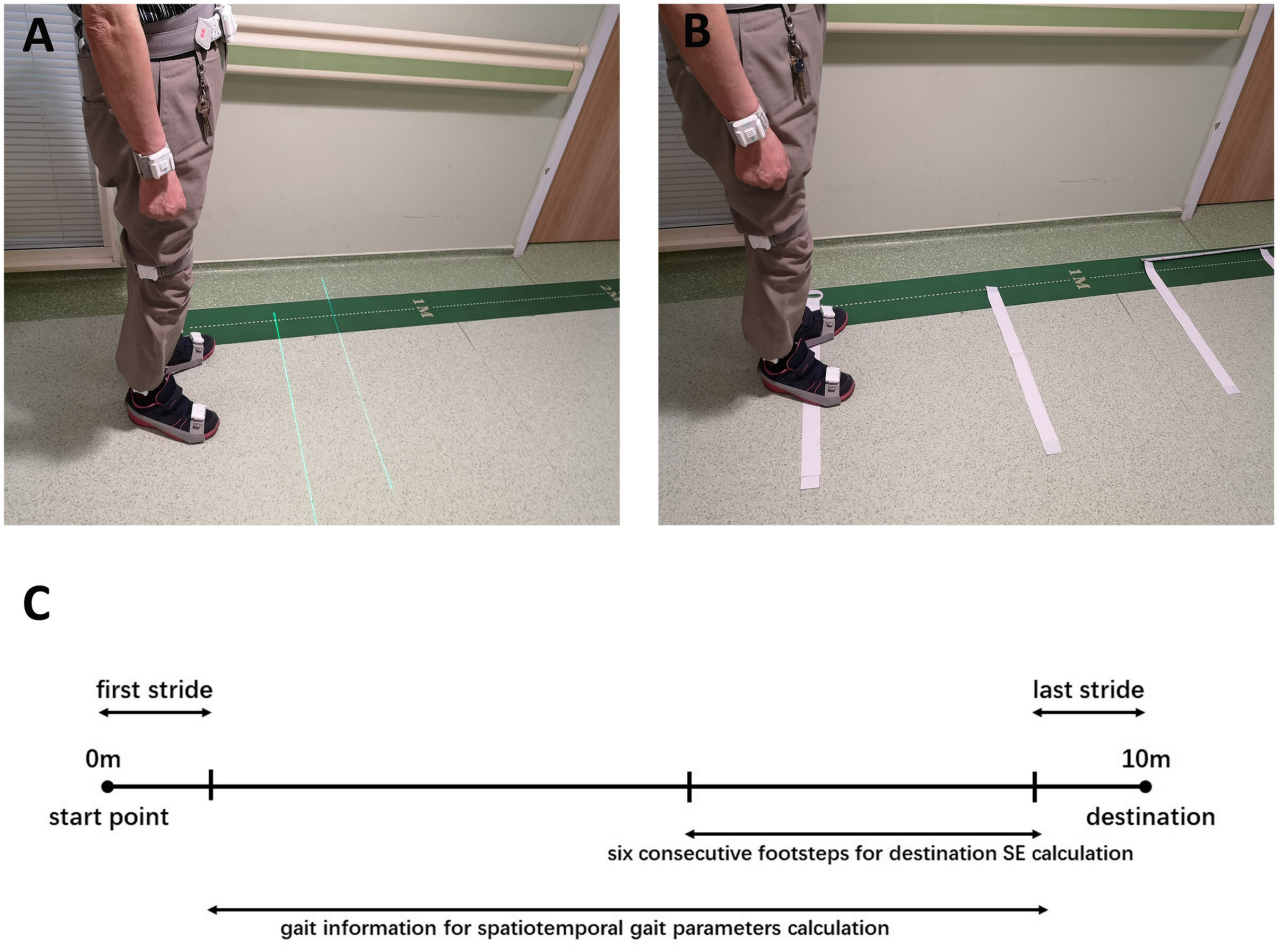
**(A)** The wearable laser lights used as visual cues. **(B)** The transverse strips used as visual cues. **(C)** Ten-meter walking trial and data used for calculating destination SE and gait parameters.

All participants received gait assessment at least 3 h after the last dopaminergic medication intake (in an end-of-dose state) (21, 22). After the investigator confirmed that each sensor was placed correctly, the participant stood still at the start point of a 10-m straight pathway and prepared. When the investigator issued the instruction, they began to walk straight, and then stopped at the end point of the 10-m distance. Each participant was guided to walk at comfortable pace. Ten-meter walking trials were tested in three conditions: no cue, laser lights and transverse strips on the floor. In the laser lights condition (Figure 1A), a laser device fixed on waist belt was used to provide two parallel transverse laser lines in front of the participant. Participants were guided to step over the laser line while walking at a comfortable pace. In the last condition (Figure 1B), transverse white strips, measuring 60 cm long and 48 mm wide, were placed on the floor with a distance in between the strips of 40% of the patient's height rounded to the nearest 5 cm, based on previous studies (21, 23). For patients whose step length was unable to be normalized, the strip intervals were set referring to their daily steps. Participants were guided to step on each strip in sequence while walking at a comfortable pace. In order to analyze the spatiotemporal parameters in a continuous gait process, the participants were required to complete each 10-m walking trial continuously without pause. If there was a freezing episode or pause during the walking, we would ask the participant to stop the experiment and have a rest. The experiment was repeated as the participant was in a better state. Three valid and analyzable trials were conducted for each walking condition, with a short break between each walking trial. If occasionally the patient was unable to complete all walking trials, each condition was only repeated twice. Walking trials in the no cue condition were always conducted first to avoid any influence from other conditions with visual cues. The remaining two conditions with different visual cues were tested in random order among the participants, thus counterbalancing the order effect.

### Gait Outcome Variables

#### Sequence Effect

The SE was measured as a regression slope, and the step to step data of each trial was extracted for further determining the slope of SE.

When calculating the regression slopes for the section of walking trials toward a destination, step length data for the six consecutive footsteps ahead of the last stride was used to avoid the influence of sharp deceleration (Figure 1C). After being numbered in sequence, the step length was plotted against step number in each walking trial, according to previous studies (11, 14, 24). The regression slopes (β), representing the sequence effect toward a destination for each individual walk, were averaged to formulate group mean average slopes, which were compiled for each condition (no cue, laser lights and transverse strips).

#### Gait Parameters

To identify spatiotemporal gait parameters in steady state for each trial, the first and last strides were excluded, avoiding acceleration and deceleration during walking (Figure 1C). For each trial, step length, step length variability, step length asymmetry, step time, step time variability, step time asymmetry, cadence, velocity, and double limb support were calculated. Left and right footstep recordings were pooled together to include more data points. Variability characteristics (e.g., step length variability and step time variability) was calculated using the coefficient of variation (CV) as CV = (SD/mean) × 100, for each trial. Asymmetry characteristics (e.g., step length asymmetry and step time asymmetry) were determined as the percentage of the average absolute difference between left and right steps for each walking trial.

These gait parameter values were averaged across three or two trials and their means combined to provide group mean data in each condition.

### Statistical Analysis

Statistical analysis was performed using SPSS 23. The differences in the study variables between the PD+FOG group and PD–FOG group, including demographic and clinical characteristics and spatiotemporal gait parameters, were assessed with Student's *t*-test and Mann–Whitney test as appropriate; *p* < 0.05 was considered significant. Satterthwaite's approximation was used for *t*-test with unequal variance. When comparing the differences in destination SE between the PD+FOG group and PD–FOG group, multiple linear regression was applied to control the baseline differences between two groups. One-way repeated-measures ANOVA was calculated to examine the differences between the three walking conditions. The *post hoc* analysis was corrected using Bonferroni correction, and *p* < 0.0167 was considered significant.

## Results

### Clinical Features

Demographic and clinical characteristics for each group can be found in Table 1. The PD+FOG group and the PD–FOG group were well-matched for age and height (*p* = 0.057 and *p* = 0.434, respectively). The UPDRS-III score (*p* = 0.007) and Hoehn and Yahr scale (*p* < 0.001) were significantly higher in the PD+FOG group than in the PD–FOG group, which may be related to the significantly longer disease duration of the PD+FOG group (*p* = 0.014). There were no significant differences in MMSE (*p* > 0.05) between the two groups, while the MoCA-B and GDS score of the PD+FOG group was significantly higher than that of the PD–FOG group (*p* = 0.011 and *p* = 0.001, respectively). As expected, the PDQ-39 score for evaluating quality of life was significantly higher in the PD+FOG group than in the PD–FOG group (*p* = 0.004).

**Table 1:** Demographic and clinical characteristics for each group.

**Group characteristics**	**PD+FOG (*n* = 15), mean (SD)**	**PD–FOG (*n* = 20), mean (SD)**	***p***
Age (years)	71.47 (6.51)	67.55 (5.25)	0.057^a^
Height (m)	1.66 (0.09)	1.68 (0.06)	0.434^a^
Disease duration (years)	8.07 (2.94)	5.50 (2.86)	**0.014**^**a**^
H&Y scale	2.53 (0.30)	1.58 (0.54)	**<0.001**^**b**^
UPDRS-III	38.27 (15.12)	26.60 (8.58)	**0.007**^**a**^
NFOG-Q	18.73 (5.75)	—	—
MMSE	27.60 (2.20)	28.25 (1.45)	0.587^b^
MoCA-B	24.80 (1.90)	26.65 (1.93)	**0.011**^**b**^
GDS	12.20 (6.57)	5.85 (3.82)	**0.001**^**a**^
PDQ-39	51.40 (35.82)	19.55 (9.84)	**0.004**^**#**^

*H&Y, Hoehn and Yahr scale; UPDRS, unified Parkinson's disease rating score; NFOGQ, New Freezing of Gait Questionnaire; MMSE, Mini-Mental State Examination; MoCA-B, Montreal Cognitive Assessment-Basic; GDS, Geriatric Depression Scale; and PDQ-39, 39-item Parkinson's Disease Questionnaire*.

*Dashes indicate where variables are not available*.

a *Student's t-test independent*.

# *Satterthwaite's approximation is used*.

b *Mann–Whitney test. Significant P-values (p < 0.05) are marked in bold*.

### Group Differences in the No-cue Condition

#### Sequence Effect

The destination SE was measured by the regression slopes (β). A negative or positive value of slope (β) represents a successive decrease or increase of the step length before reaching the destination, respectively.

**Figure 2:**
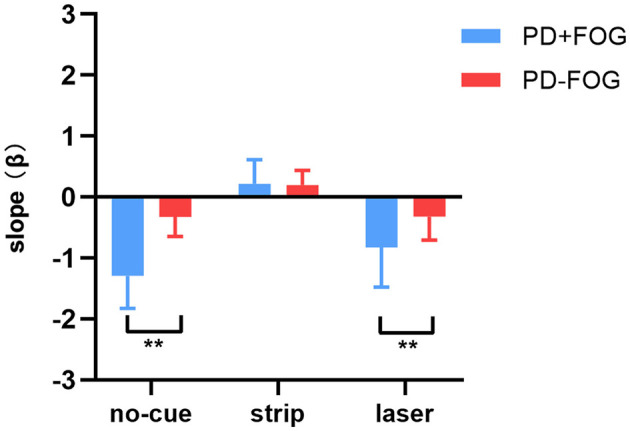
Differences in destination sequence effect (represented by β values) between the PD+FOG group and the PD–FOG group across three conditions (** *p* < 0.01).

The PD+FOG group had greater absolute β values than the PD–FOG group (Table 2). In the no-cue condition, both groups had negative β values. Using clinical features (disease duration, UPDRS-III, H&Y) and gait parameters (step length and step length variability) as covariates in the analysis, the PD+FOG group demonstrated a significantly higher absolute β-value compared to the PD–FOG group (PD+FOG, −1.29 ± 0.54; PD–FOG, −0.33 ± 0.32; *p* < 0.001) (Figure 2).

**Table 2:** Summary of average slope (β) values for the two groups across conditions for each group.

**Group**	**Condition**		
	**No-cue, mean (SD)**	**Strip, mean (SD)**	**Laser, mean (SD)**
PD+FOG (*N* = 15)	−1.29 (0.54)	0.22 (0.39)^*^	−0.83 (0.65)
PD–FOG (*N* = 20)	−0.33 (0.32)	0.20 (0.24)^*^	−0.32 (0.38)

* *Significant difference from all other conditions (p < 0.0167)*.

#### Gait Dynamics

Spatiotemporal gait characteristics in the no-cue condition for each group are shown in Table 3. Without visual cues, the PD+FOG group had significantly shorter step length (PD+FOG, 48.04 ± 15.08 cm; PD–FOG, 60.87 ± 5.35 cm; *p* = 0.006), slower velocity (PD+FOG, 0.84 ± 0.28 m/s; PD–FOG, 1.06 ± 0.11 m/s; *p* = 0.009), greater step length variability (PD+FOG, 6.73 ± 4.35%; PD–FOG, 2.88 ± 0.60%; *p* = 0.004) and asymmetry (PD+FOG, 1.30 ± 1.36%; PD–FOG, 0.48 ± 0.20%; *p* = 0.037) compared with the PD–FOG group. No significant differences were found in other gait parameters between the two groups in the no-cue condition.

**Table 3:** Spatiotemporal characteristics of gait in the no-cue condition for each group.

**Spatiotemporal variables**	**PD+FOG (*n* = 15), mean (SD)**	**PD–FOG (*n* = 20), mean (SD)**	***p***
Step length (cm)	48.04 (15.08)	60.87 (5.35)	**0.006**^**#**^
Step length variability (%)	6.73 (4.35)	2.88 (0.60)	**0.004**^**#**^
Step length asymmetry (%)	1.30 (1.36)	0.48 (0.20)	**0.037**^**#**^
Step time (s)	0.56 (0.08)	0.55 (0.04)	0.708
Step time variability (%)	7.40 (7.32)	5.06 (2.12)	0.182
Step time asymmetry (%)	9.55 (8.70)	6.82 (4.00)	0.224
Cadence (steps/min)	111.35 (13.62)	110.28 (8.45)	0.777
Velocity (m/s)	0.84 (0.28)	1.06 (0.11)	**0.009**^**#**^
Double limb support (%)	22.93 (6.78)	19.72 (3.15)	0.106^#^

*p-values are determined by t-test*.

# *Satterthwaite's approximation is used. Significant p values (p < 0.05) are marked in bold*.

### Effects of Different Visual Cues

#### Sequence Effect

After using transverse strips on the floor, the absolute β values in the two groups both decreased; there were no significant differences of the positive β values between the two groups (PD+FOG, 0.22 ± 0.39; PD–FOG, 0.20 ± 0.24; *p* = 0.844) (Figure 2). In contrast, using wearable laser lights failed to decrease the β values in the two groups, and the absolute β value in the PD+FOG group remained significantly greater than that in the PD-FOG group (PD+FOG, −0.83 ± 0.65; PD–FOG, −0.32 ± 0.38; *p* = 0.007) (Figure 2). These comparisons had already taken into account the between-group differences in clinical features (disease duration, UPDRS-III, H&Y) and gait parameters (step length and step length variability).

When comparing within each group (Table 2), the β values were significantly different across three walking conditions in both PD+FOG group [*F*_(2,28)_ = 56.884, *p* < 0.001] and PD–FOG group [*F*_(2,38)_ = 21.511, *p* < 0.001]. Within each group, *post hoc* tests revealed that the β values of the strip condition were significantly reduced compared to the other two conditions, while there were no significant differences between the no-cue and the laser conditions.

**Figure 3:**
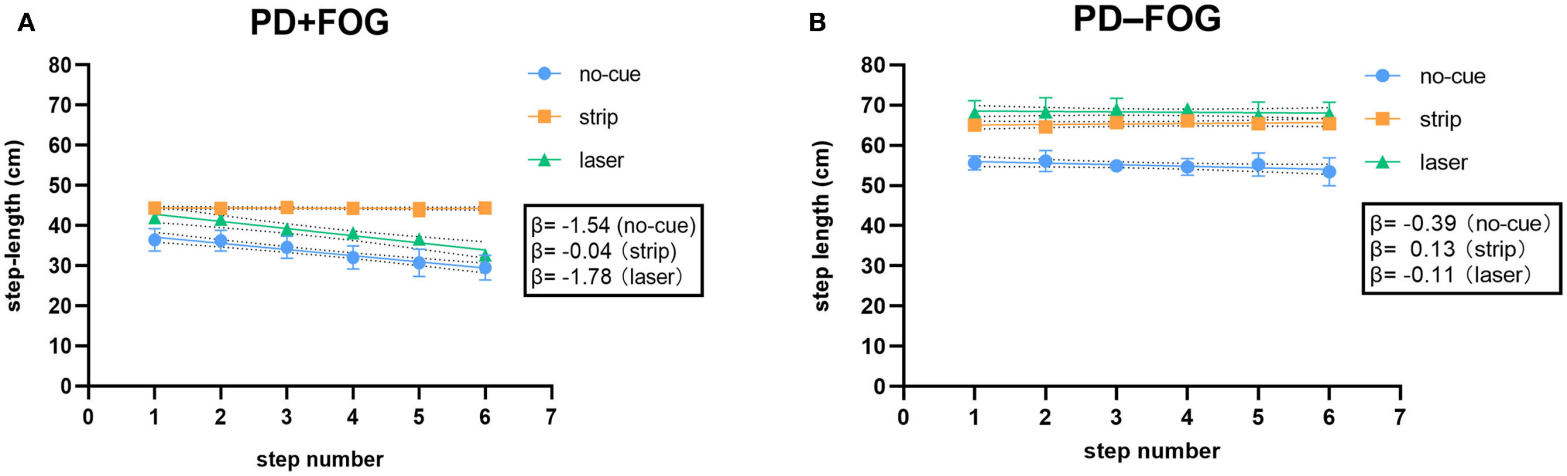
**(A)** Relationship between step length and step number for a single PD+FOG participant toward a destination in the no-cue, strip and laser conditions. **(B)** Relationship between step length and step number for a single PD–FOG participant toward a destination in the no-cue, strip and laser conditions. (Dotted area behind the linear regression line represents 95% confidence bands).

For a single age-matched individual, the PD+FOG participant (Figure 3A) had negative and steeper slopes in both no-cue (β = −1.54) and laser (β = −1.78) conditions, indicating that the marked SE occurred before reaching the destination. In contrast, the PD–FOG participant had negative but relatively flat slopes in both no-cue (β = −0.39) and laser (β = −0.11) conditions (Figure 3B), indicating the presence of the mild SE toward the destination. For each participant, destination SE was improved only in the strip condition (PD+FOG, β = −0.04; PD–FOG, β = 0.13), while step length was increased in both strip and laser conditions.

#### Gait Dynamics

**Figure 4:**
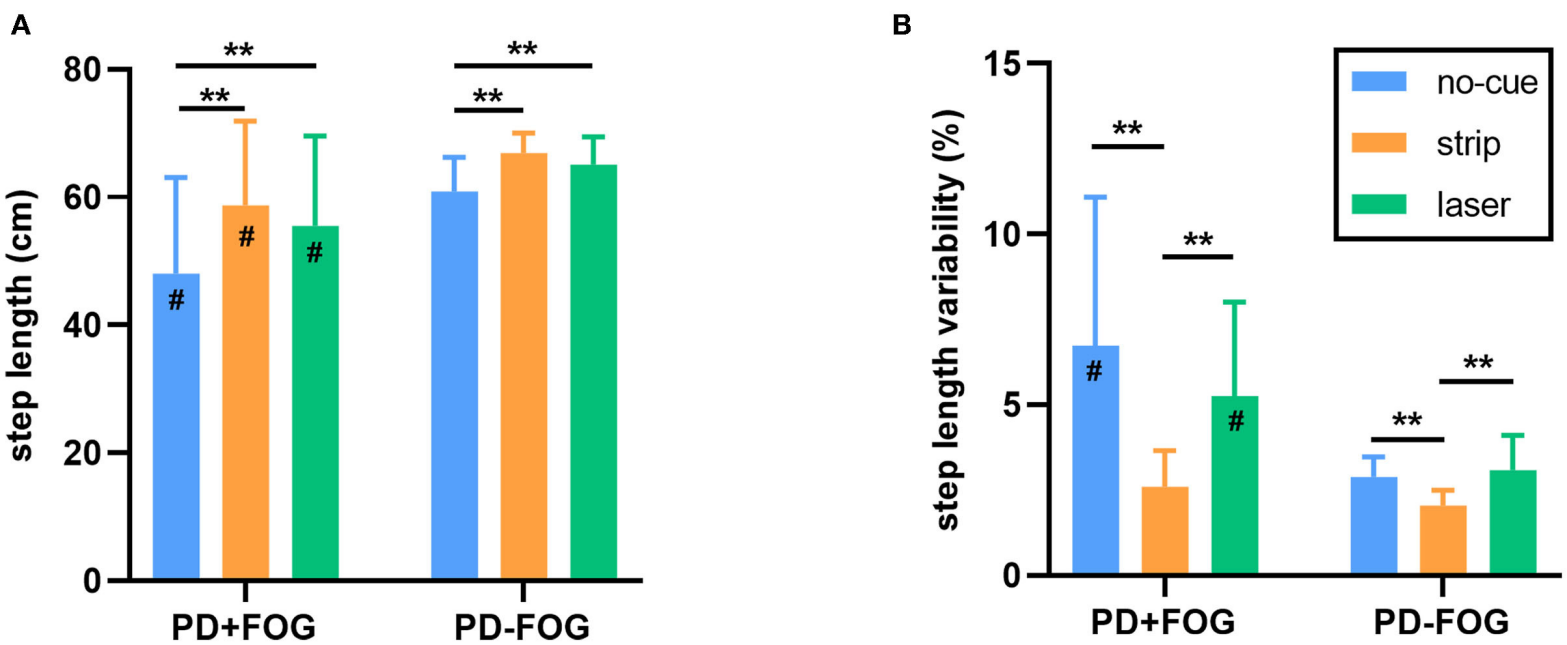
**(A)** The step length of each walking condition in the PD+FOG group and the PD–FOG group. **(B)** The step length variability of each walking condition in the PD+FOG group and the PD–FOG group. [**p < 0.01, ^#^significantly different than the PD–FOG group (*p* < 0.05)].

Both visual cues improved gait parameters. There were significant differences in step length across conditions for both the PD+FOG group [*F*_(2,28)_ = 14.877, *p* < 0.001] and the PD–FOG group [*F*_(2,38)_ = 18.329, *p* < 0.001] (Figure 4A). *Post hoc* tests determined the differences in step length between the no-cue condition and the other visual cue conditions. Step length was increased significantly with either of the visual cues (both *p* < 0.01). In addition, the step length variability also differed significantly across conditions for both the PD+FOG group [*F*_(2,28)_ = 13.861, *p* < 0.001] and the PD–FOG group [*F*_(2,38)_ = 15.861, *p* < 0.001] (Figure 4B). *Post hoc* tests confirmed that the step length variability in the strip condition was significantly smaller than that in the no-cue and laser conditions (both *p* < 0.01).

## Discussion

This study determined that PD patients who experienced FOG displayed much greater SE before approaching a destination. Both visual cues could improve gait parameters. However, only the transverse strips on the floor could alleviate destination SE in PD patients.

Our study found that the destination SE was more severe in the PD+FOG group than in the PD–FOG group, which indicates that PD patients with FOG could exhibit more progressive decrease in step length toward their destination. Step length reduction and occurrence of SE have been proposed to be dual requirements for inducing FOG (11, 13). Therefore, motor blocks will not occur in the absence of SE. In fact, motor blocks can be induced with even larger step length, because the effect of SE can be much greater in some circumstances than in others. In other words, if the SE is great enough and able to get command of stepping, the steps will become smaller and smaller until a motor block occurs. In addition to the significant destination SE, the step length of the PD+FOG group was also smaller in this study. Therefore, our findings could make proper interpretation of why FOG patients are likely to freeze when approaching their destination.

The mechanism of destination SE in PD patients could be explained by the concept of BG function defects in running automatic movement (11, 13). In conjunction with the supplementary motor area (SMA), the BG runs automatic movement by maintaining motor set and providing timing cue. In PD patients, the timing cues are disrupted, thus leading to the SE (13). The differences in destination SE between the two groups might be related to the differences in degree of BG function injury, and the PD+FOG group may be more severely injured. Similarly, there was a successive decrease in step length (SE) prior to turning or when passing a doorway in PD patients, and a significantly greater decline in step length was observed in the FOG patients (15, 16). Due to impaired automation, gait control is often dependent on attention, especially in PD patients with FOG. Overall, destination, turning and doorway are well-known environmental factors that can trigger FOG in PD patients (3). These variable environments could be distracting, and then the stepping might switch from attention to uncompensated automatic control. Therefore, FOG could be induced by the presence of SE and reduced step length.

Wearable laser lights and transverse strips on the floor had disparate effects on the destination SE. Both visual cues could increase step length. However, only the transverse strips on the floor could alleviate destination SE. As noted earlier, FOG during walking will not occur unless the SE is present (13). Increasing background step length would make the SE less significant, thereby reducing the likelihood of freezing (11, 14). Transverse lines on floor are well-known strategies to reduce FOG (25–27). In recent years, wearable laser lights have been designed to deal with FOG in PD patients. However, wearable laser lights failed to rescue FOG in some previous studies (28–30). Our results might explain this variability properly. Laser lights could increase step length but fail to alleviate the SE. Therefore, if the SE is significant enough and exceeds the compensation, FOG will be induced (13). In contrast, transverse strips on the floor could not only increase step length, but also alleviate the SE, thus greatly reducing the risk of FOG. Our findings are similar to those of Iansek et al. who observed that medication, attentional strategies and visual cues all improved hypokinesia, whereas only visual cues, in the form of transverse white strips on the floor, were able to eliminate the SE (11). Strategies that can eliminate SE are likely to be the only rescue plan for on-state freezing. Compared to wearable laser lights, the strips on the floor are not portable. Strips on the floor may be more suitable for indoor FOG rehabilitation, while wearable laser lights offer the potential for alleviating freezing in daily activities. A recent research showed that pavement patterns designed in the form of large transversal visual cues could help improve gait in PD patients (27), and this may be a feasible strategy.

The slope of destination SE could be visualized in a scatterplot. Since linear regression of all subjects in one graph might complicate the results, the scatterplot for a single age-matched individual in each group was presented (Figure 3). This could make the main findings on destination SE easier to understand. In the no-cue condition, the slope of the PD+FOG participant (Figure 3A) was steeper than that of the PD–FOG participant (Figure 3B), which is consistent with the group findings that PD patients with FOG displayed much greater SE before approaching a destination. For each participant, destination SE was improved only in the strip condition, while step length was increased in both strip and laser conditions. These were also consistent with the group findings.

In this study, the PD+FOG group had greater step length variability and asymmetry during baseline walking, which is a hallmark feature of gait instability. The results are consistent with other researches on gait analysis of PD patients (31, 32). The presence of SE is often accompanied by greater step length variability (11, 14). We also observed a similar phenomenon that the PD+FOG group exhibited both greater destination SE and step length variability. Gait variability measures have received great attention in PD and disease progression (33, 34). In our results, step time variability, step time asymmetry and double limb support were higher in the PD+FOG group but did not reach statistical significance. Despite this, double limb support was reported significantly higher in PD patients than in the age-matched healthy control group (31, 35). When the PD+FOG group walked with a shorter step length and longer step time in the no-cue condition, they would naturally walk at a significantly slower speed.

In line with previous studies (14), the PD+FOG group had significantly higher UPDRS motor scores, H&Y scales and NFOG-Q scores, along with longer disease duration. It is reported that in early stages of PD, between 21 and 27% of patients experience freezing, while this number rises up to 80% in the advanced stages (36). As anticipated, advanced PD patients could have more severe motor performance and higher H&Y scales. Depression is considered to be related to FOG in PD patients (37). Our results consistently showed that the PD+FOG group had mild depression on average, while the PD–FOG group had a relatively normal GDS scores. The FOG patients suffered from disturbing symptoms, and as a result, their quality of life was severely impaired.

There are some limitations in current study. First, while the two groups were matched for age and height, there was a considerable difference in the reported clinical features (disease duration, UPDRS-III and H&Y scores) and gait parameters (step length and step length variability) in the no-cue condition. However, multiple regression analysis suggests the slopes are independent of these variables which supports the conclusion that FOG patients had significantly greater destination SE than PD patients without FOG. Second, to ensure the safety of the participants, our study was investigated in end-of-dose state instead of off-state. FOG patients might present greater SE but easily fall in the off-state. Third, due to this relatively small sample size, actual freezing episodes were not involved in the analysis.

## Conclusion

In summary, this study demonstrated that PD patients with FOG presented significantly greater destination SE compared to PD patients without FOG. These findings might explain why FOG patients tend to freeze when they reach their destination. Both the transverse strips on the floor and the wearable laser lights are able to increase step length. However, only the transverse strips can alleviate destination SE. Therefore, visual cues using transverse strips on the floor might be a more effective strategy for FOG rehabilitation.

## Data Availability Statement

The raw data supporting the conclusions of this article will be made available by the authors, without undue reservation.

## Ethics Statement

The studies involving human participants were reviewed and approved by the Medical Ethics Committee of Tongren Hospital, Shanghai Jiao Tong University School of Medicine. The patients/participants provided their written informed consent to participate in this study.

## Author Contributions

S-SC contributed to the conception of the study, data acquisition, and writing of the first draft. X-PW contributed to the design and organization of the work, manuscript review, and critique. X-ZY, S-HW, and RT contributed to the data acquisition, manuscript review, and critique. All authors read and approved the final manuscript.

## Conflict of Interest

The authors declare that the research was conducted in the absence of any commercial or financial relationships that could be construed as a potential conflict of interest.
